# Refining the evidence linking dietary diversity, genetic susceptibility, and dementia

**DOI:** 10.1016/j.tjpad.2025.100247

**Published:** 2025-06-20

**Authors:** Hongye Yao, Yangbo Lv

**Affiliations:** a The Second School of Clinical Medicine, Zhejiang Chinese Medical University 310053 Hangzhou, PR China; b Department of colorectal surgery, The Quzhou Affiliated Hospital of Wenzhou Medical University, Quzhou People's Hospital 324000 Quzhou, PR China

To the Editor:

We read with great interest the article by Zhao et al., titled “Association of dietary diversity, genetic susceptibility, and the risk of incident dementia: A prospective cohort study [1].” This large-scale and methodologically rigorous study, leveraging the UK Biobank cohort, offers valuable insights into the potential protective role of dietary diversity in dementia risk, particularly among individuals with higher genetic susceptibility. While the study has many strengths, including comprehensive adjustments for confounders and stratified analyses, we would like to highlight several areas where further refinement may strengthen the interpretation of the findings.

First, the authors used Cox proportional hazards models to assess associations between dietary diversity, polygenic risk scores (PRS), and incident dementia. However, this approach may underestimate risk in the presence of competing events, particularly death [2]. To provide more accurate risk estimates, we suggest performing sensitivity analyses using competing risk models in which death is treated as a competing event. Such models would offer a clearer understanding of the cumulative incidence of dementia in older populations.

Second, although the UK Biobank provides an exceptionally rich dataset, it is important to acknowledge the inherent selection bias associated with volunteer-based cohorts. Only 5.5 % of those invited to participate in the UK Biobank enrolled, and participants tend to be healthier, better educated, and more likely to engage in favorable lifestyle behaviors than the general UK population [3,4]. This selection bias may substantially affect the estimation and generalizability of the lifestyle-dementia associations, particularly those involving the composite healthy lifestyle score.

Third, the study defined dementia as a binary outcome based on clinical diagnosis. While this is standard practice, it may not fully capture the nuanced relationship between dietary diversity, genetic risk, and early neurodegenerative changes. The UK Biobank contains valuable peripheral biomarkers—such as plasma phosphorylated tau (P-tau), neurofilament light chain (NfL), and glial fibrillary acidic protein (GFAP)—that could be leveraged to evaluate subclinical cognitive decline or neurodegeneration [5]. Including such intermediate phenotypes in future analyses would enhance the mechanistic understanding of how dietary diversity and genetic risk jointly influence the dementia trajectory.

In conclusion, Zhao et al. provide compelling evidence supporting dietary diversity as a modifiable factor in dementia risk, especially among those with elevated genetic predisposition. We commend the authors for their contribution and encourage future research that further refines these findings through mechanistic biomarkers, competing risk frameworks, and population-representative validation.

## CRediT authorship contribution statement

**Hongye Yao:** Conceptualization, Writing – original draft, Writing – review & editing. **Yangbo Lv:** Funding acquisition, Supervision.

## Declaration of competing interest

The authors declare the following financial interests/personal relationships which may be considered as potential competing interests: Yangbo Lv reports financial support was provided by People’s Hospital of Quzhou. If there are other authors, they declare that they have no known competing financial interests or personal relationships that could have appeared to influence the work reported in this paper.

## References

[1] Zhao B., Cheng B., Li X., Xia J., Gou Y., Kang M., Hui J., Liu Y., Zhou R., Liu C. (2025). Association of dietary diversity, genetic susceptibility, and the risk of incident dementia: a prospective cohort study. J Prev Alzheimers Dis.

[2] Wolbers M., Koller M.T., Stel V.S., Schaer B., Jager K.J., Leffondre K., Heinze G. (2014). Competing risks analyses: objectives and approaches. Eur Heart J.

[3] Stamatakis E., Owen K.B., Shepherd L., Drayton B., Hamer M., Bauman A.E. (2021). Is cohort representativeness passé? Poststratified associations of lifestyle risk factors with mortality in the UK Biobank. Epidemiology.

[4] Davis K.A., Coleman J.R., Adams M., Allen N., Breen G., Cullen B., Dickens C., Fox E., Graham N., Holliday J. (2020). Mental health in UK Biobank–development, implementation and results from an online questionnaire completed by 157 366 participants: a reanalysis. BJPsych Open.

[5] Wang X., Shi Z., Qiu Y., Sun D., Zhou H. (2024). Peripheral GFAP and NfL as early biomarkers for dementia: longitudinal insights from the UK Biobank. BMC Med.

